# Experiences of women with impaired physical mobility during pregnancy, childbirth and postpartum: A case study

**DOI:** 10.18332/ejm/170433

**Published:** 2023-10-05

**Authors:** Marika Merits, Kadi Lubi, Meelike Tammes

**Affiliations:** 1 Midwifery Department, Health Education Centre, Tallinn Health Care College, Tallinn, Estonia; 2 Department of Health Technologies, School of Information Technologies, Tallinn University of Technology, Tallinn, Estonia

**Keywords:** impaired physical mobility, pregnancy, childbirth, postpartum period, maternity care

## Abstract

**INTRODUCTION:**

The research deals with a little-studied topic in Estonia: the experiences of women with impaired physical mobility (IPM) during pregnancy, childbirth, and in the postpartum period. Women with IPM, a vulnerable group, have a higher risk of complications and a higher probability of missing out on comprehensive maternity care.

**METHODS:**

The method of the present research is qualitative. It is a case study of three women with IPM with whom semi-structured interviews were conducted.

**RESULTS:**

It was found that women with IPM encountered several obstacles and problems during pregnancy, childbirth, and the postpartum period; despite this, women’s experiences with maternity care were mostly positive. Furthermore, there are several areas for improvement to ensure more comprehensive maternity care. Women with IPM need more support and help, and midwives are expected to have additional knowledge regarding the specifics or limitations resulting from mobility impairments.

**CONCLUSIONS:**

In the future, the topic needs greater attention and improvement in the Estonian context to ensure more comprehensive maternity care for women with IPM. It is important to provide midwives the knowledge and skills to assist women with IPM during pregnancy, childbirth, and the postpartum period.

## INTRODUCTION

According to the WHO, the definition of ‘disability’ includes impairment of body function or structure, limitation of movement in the performance of various activities, and limitation of participation in several life situations. Disability cannot be seen as just a health problem. It is a complex phenomenon that reflects the interaction between the human body and society. For people with disabilities to be able to cope more independently with the activities of everyday life, societal innovations and the removal of environmental and social barriers are needed^1^. Physical disability can arise from congenital causes (e.g. spina bifida, cerebral palsy, etc.), various traumas acquired during life (e.g. spine injuries), or other medical conditions (limb amputation, tumors, etc.)^2^.

Women with impaired physical mobility (IPM) have a higher risk of complications during pregnancy, childbirth, and the postpartum period than women without disabilities^3^. Women with disabilities are more likely to miss out on the maternity care services they need because access to health facilities and services is compromised. Also, women with IPM may perceive that the maternity care provided to them does not meet their expectations and that the health professionals’ lack of knowledge puts both their health and the health of the fetus at risk^4^. Previous studies conducted among women with IPM have shown that, according to women, there is little evidence-based information for them^5,6^, the attitude of specialists is problematic^7-9^ and their knowledge about the functional specificities related to mobility impairment is superficial^5,10,11^.

Furthermore, several studies have confirmed that health workers, including midwives, lack knowledge and skills in helping women with mobility disabilities during pregnancy, childbirth, and the postpartum period. It is emphasized that there is not enough training and practice to provide maternity care to women with mobility impairments^12,13^.

According to the database of Statistics Estonia, as of 2022, there were 146162 disabled people in Estonia or about 11% of the total population. Of these, 9871 were women of reproductive age (16–54 years)^14^. According to the Estonian Chamber of Disabled People (EPIK), the number of women with IPM is on the rise, which is due to early and more effective diagnosis^11^.

The present study is important because women with IPM are a vulnerable group, and the experiences of women with IPM during pregnancy and childbirth and in the postpartum period have not yet been discussed in Estonia. Previously, a study related to IPM in Estonia was published in 2002 in the journal *Eesti Arst* (Estonian Doctor), where the pregnancy and postpartum risks arising from multiple sclerosis and the importance of organizing possible support systems were described^15^. For clarification, it is added that registered midwives work in women’s clinics and hospitals in the field of maternity care in the Estonian healthcare system. In addition, social workers and care nurses also work in the field of maternity care to ensure more comprehensive care.

## METHODS

### Study design

The method of the present research is qualitative. It is a case study because it is an appropriate way to describe human experiences and attitudes in a situation where there are few subjects, and therefore convenience sampling is used^16^. Convenience sampling is used because the inclusion of subjects in the study is difficult due to the sensitivity of the topic and the specificity of the target group, etc.^17^. Three women with IPM with whom interviews were conducted participated in the study. The women were aged 37–45 years, and all women had a partner and an average of two children. One subject has been in a wheelchair since adolescence, and the other two use crutches and/or supporting armrests for movement. The criteria for inclusion in the study were IPM, reproductive age, and childbirth that took place within the last ten years. The time limit for giving birth was set to ten years because, in the case of previous pregnancies and births, the conditions and possibilities may differ too much from the currently functioning system. To find subjects who met the criteria of the present study, the authors cooperated with the Estonian Union of Persons with Mobility Impairment (ELIL) which forwarded an invitation to participate in the study to potential participants.

### Data collection

The interviews took place between 1 July 2020 and 14 October 2020. The interviews were conducted by telephone to provide an anonymous environment for the research subjects and to consider limitations due to mobility impairments. Three women with IPM with whom semi-structured interviews were conducted participated in the study. The interviews were recorded and later transcribed verbatim. The length of the interviews varied between 25 and 36 minutes. An introduction to the study, an interview schedule, and an informed consent sheet were sent electronically to the interviewees in advance for perusal. Qualitative content analysis was used to analyze the transcribed interviews. Qualitative content analysis is a text analysis that considers the background in which systematic rules are followed for coding the meanings and content of the text^18^.

### Statistical analysis

**Figure 1:**
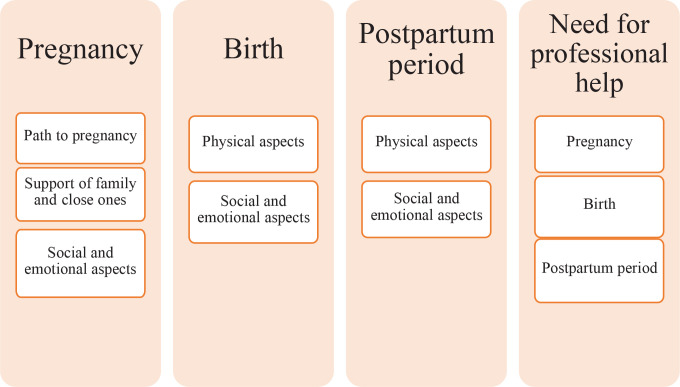
The formation of categories and codes

The present study was based on the experiences of women with IPM, and the context related to maternity care during pregnancy, childbirth, postpartum period (hospital and at home) and the need for professional help. During the content analysis of the text, a scheme for the formation of categories and codes was formed (Figure 1).

As can be seen in Figure 1, four main categories were formed: pregnancy, childbirth, postpartum period (in-patient and home setting), and the need for professional help. Codes emerged as the result of the content analysis. The pregnancy category consisted of the following codes: the journey to pregnancy, support from family and loved ones, and social and emotional aspects. Birth and postpartum categories include physical as well as social and emotional aspects. The fourth category is the need for professional help, which involves aspects of maternity care.

### Ethics considerations

The study has been approved by the Ethics Committee (TAIEK) for Human Research of the Institute for Health Development (TAI) by decision No. 2635 (21.02.2019). In addition, the consent of the research subjects is required to conduct research. Participation in the research was voluntary for the participants. Interviewees had the right to withdraw from participating in the research at any stage of the research. Subjects digitally signed an informed consent form before the start of the study and were introduced to the objectives, methodology, and benefits of the research in advance. Study participants were informed about the use, storage, and destruction of data.

## RESULTS

In interpreting the results, it was considered that the aspects that emerged were discussed in different interviews and were not one-time expressions of opinion. The most eloquent examples are presented as quotations, not all expressions of the same topic.

### Experiences of women with IPM during pregnancy

The interviewees’ quotes intertwine the journey to pregnancy, aspects related to support from loved ones, as well as social and emotional aspects.

The women in the study have had different journeys to pregnancy. According to one of the interviewees, her age and finding a life partner became decisive when planning a pregnancy. Also, the woman had always wanted to have children:

*‘Well, yea. I have always wanted pregnancy and children, and there was the pressure of my age also, and then I got myself a partner, and then I decided … that (pause), to have a child.’* (I1)

Another interviewee’s journey to pregnancy was more difficult and at the same time very emotional for her, and she had a couple of miscarriages before the desired pregnancy. She could not point out a direct connection between her illness and spontaneous abortions. She already had two adult children and had developed the mobility impairment after their birth:

*‘Well, I can’t say whether it’s with this disease, but there have been spontaneous interruptions there, yes, kind of. There have been a few times, yes.’* (I3)

*‘All this was very difficult for me emotionally and I could not have coped without the support of my loved ones.’* (I2)

At the same time, the woman had a previous traumatic experience with the death of an intrauterine fetus. According to the woman, she was eight months pregnant at that time. Doctors attributed the incident to high blood pressure:

*‘Because I’ve lost a baby once, er, also basically like, er, I was already in my eighth months, huh? And then because the blood pressure was so high and like the baby was stillborn. That then there was, well, like there was already a sort of fear about it.’* (I3)

The previous quotes show that the journey to pregnancy was different for women, and the experience of conception and pregnancy is not always related to the presence of a disability. All the above-mentioned experiences can be traumatic and affect a woman’s quality of life.

One interviewee pointed out that the midwife did not accept the friend she brought to the appointment as a support person. The woman realized that her friend was not welcome there and thereafter did not ask her to come with her. This situation was emotionally difficult for the interviewee:

*‘You know, for the first time, I took a friend with me as a support person. When my pregnancy was confirmed, I was met with a kind of disapproval like who is this person? Why is she here? That, well, well, it was like that, but since I was able to handle it myself in the end … so I didn’t take her with me anymore.’* (I1)

This quote illustrates the limitations of the social and emotional aspect. In the quote, the girlfriend’s suitability as a support person was ruled out, and her presence during the appointment was questioned, which leaves women in an uncomfortable situation because, in fact, everyone has the right to bring a person who is safe for them, who does not necessarily have to be a male partner. The possible condescending attitude of a midwife can reduce the sense of emotional security of pregnant women. As the support persons are mostly the women’s male partners, the difference from the usual situation can be unusual for midwives or other healthcare professionals.

However, the interviewees had good experiences where the midwife was very open and supportive. The interviews revealed that women had a fear of prejudice on the part of health professionals, but the experience was the opposite:

*‘That, uh, (sigh), mmm, well, some kind of moment, I say that uh, like maybe, in the beginning, I was afraid that there were bigger, well, I was just afraid of those prejudices, well, for example, somewhere, well, at the gynecologist or in the maternity hospital or, or at such special services, the attitude there was actually very open and, and even I myself had perhaps bigger prejudices than the people who worked there.’* (I2)

*‘The social worker who met me did comment that how am I going to cope when I myself have difficulties.’* (I1)

As the quote shows, the fear of negative attitudes from midwives or other healthcare workers was unfounded, but at the same time, negative attitudes from social workers were experienced.

All interviewees had a supportive network, which is important when starting a family. The main helpers daily were the women’s partners/husbands. According to one interviewee, she tried to conceive for years. When she finally got pregnant, her husband was a great support and helped her:

*‘… (supported) very much, helped all the time that uh, like because we both wanted it very much … or well, well we wanted it and, and it wasn’t, uh, easy in the sense that, that it took time, this conception itself … that we tried for years, er, we kept trying and trying and nothing, then, er, well, then, he really, yes.’* (I2)

In addition, the women were assisted by their other family members. In most cases, helping was based on what the woman needed. According to one woman, she was mostly able to manage on her own, but when she needed help, her family was there for her:

*‘… (pause). Well. (pause) (laughter). I can’t even say, well, they help me with everything when I ask, uh … (pause). I can’t even say.’* (I1)

The quotes show that a support network plays a big role in creating and maintaining a family. Family support is important for both healthy and disabled women. Women with IPM need support and help more than usual, including more social and emotional support and a functioning support network is decisive in the decision to have children.

The experiences of the participants in getting pregnant and during pregnancy were different, and there were both positive and negative experiences. Interviewees had mainly pleasant and better-than-expected experiences with midwives during pregnancy, except for one woman. The interviewees had a functioning support network, both in the form of a partner and family.

### Experiences of women with IPM during childbirth

This topic dealt with childbirth and the impact of mobility impairments on childbirth and the physical, social, and emotional aspects involved. Two women gave birth by planned cesarean section, and one by vaginal delivery. One of the women was aware that she would not have been able to give birth on her own, and a cesarean section was the right choice in this situation:

*‘No, I had no other expectations because I know that I would have never been able to cope with the childbirth on my own, that my body is too weak for it, to put myself and my child in such a situation that … there was no point.’* (I1)

*‘But I was so nervous and emotionally completely exhausted, it was still a huge tension (pause), you still expect something positive (pause) and there was a great fear that hopes might be dashed.’* (I2)

*‘In fact, it was both physical and emotional stress, but the desire to give birth and become a mother was overwhelming.’* (I1)

These quotes show clearly how much emotional stress a woman has and how important it is to provide empathic support in a critical situation.

The third interviewee gave birth vaginally. Her labor was induced due to low amniotic fluid:

*‘Well, basically, the birth was induced with a drip like, uh, because in the last ultrasound, they saw that there was little amniotic fluid, so it was like induced. But it was born kind of by itself and quite quickly, and, like, nothing was wrong with me.’* (I3)

*‘Yes (pause), physically I was a bit troubled (laughter).’* (I3)

Two out of three women gave birth by cesarean section, and one by vaginal delivery. Pain relief methods for each delivery and cesarean section were different. All experiences were different, but the quotes show that a human-centered approach was experienced during childbirth, where abilities and wishes were considered. Unfortunately, not enough attention was paid to the emotional tensions of the woman, therefore both social and emotional support was limited.

### Experiences of women with IPM in postpartum period

This topic dealt with the postpartum period in the hospital and at home.

Women stayed in the hospital for about four to six days after giving birth. According to the participants, the postpartum period passed without complications:

*‘Well, because the boy’s blood sugar dropped, we were in the hospital for six days. I was fine, but because the boy was in intensive care, er, because his blood sugar was very low, we were there like six days. But then they let us go home, and then everything was fine.’* (I3)

As the quote shows, longer hospital stays for women with IPM may not always be related to a health condition stemming from the disability.

Due to mobility impairments, one of the interviewees was allowed to stay in the family room after a cesarean section, but problems were caused by the inability of the hospital’s nursing staff to assist the woman after childbirth:

*‘Well, what about the postpartum period in the hospital, the five days we were there, it was very, very nice, of course, that we could stay in the family room because the general room didn’t have an accessible toilet at all, that we also got a separate room, that we had a toilet and … that it was very nice, but at the same time, those from the hospital … these care workers, well, in their case, I have to say that they were not ready for a patient like me.’* (I2)

*‘Of course, I would like to manage by myself so that I don’t have to ask for help, but … (pause). Embarrassing thing. So, it is.’* (I2)

As it was already evident earlier, the problems are not so much experienced by midwives but rather by social workers and care nurses, which is why healthcare institutions need to pay attention to this aspect to enable a comprehensive human-centered approach to the woman giving birth and their families during the entire period of their stay in the hospital, regardless of their health status. As a bottleneck, the lack of specific toilets hinders a woman’s physical coping, with accompanying social and emotional problems.

Another interviewee had a completely different experience. She never once felt that no one wanted to deal with her during her stay in the hospital:

*‘… (pause). Well, no, I didn’t experience that, no. Or I didn’t have the feeling that er … that they didn’t want to deal with me.’* (I1)

In summary, it can be said that all interviewees stayed in the hospital longer than average. The average postpartum hospital stay in 2020–2022 in Estonia was 2.8 days^19^. The reasons were related to both the needs arising from the cesarean section and, in one case, the child. The interviewees expected understanding, emotional support, and information. They expected the midwives to be able to carry out normal postnatal and disability-related counseling.

After giving birth, various aids and learned techniques on how to hold, lift, place, and transport the baby more safely, played an important role for women at home. For all the interviewees, holding the child in their arms was problematic to some extent. For one woman, holding her baby in her arms and moving with her like this was scary because she had previously fallen during her pregnancy:

*‘Well, holding the baby at all, holding it in your arms and that, that was scary for me.’* (I1)

According to one of the interviewees, she had no problems after giving birth, as she was able to move freely with the child without help. Due to her place of residence and a functional support system, she was able to cope with her daily activities:

*‘(loud sigh) I don’t, I can’t say, I don’t know. I was like, very well, I don’t know. That, er, well, I don’t know what to say. I lived in a country house, in the sense that these, er, wood and things were brought to me by my husband, that I could handle the child well myself, er … that, well, that when I went for a walk with him, it was very good, I did not have this backache and …’* (I3)

The same interviewee was asked what kind of help she would have needed from healthcare professionals after childbirth. Because she had a good support system and knowledge, and was able to handle everyday activities herself, it was a bit difficult for her to put herself in the shoes of others. According to the woman, the need depends on the person and her state of health:

*‘(pause) I can’t say, because, well, I don’t know, I didn’t really need a lot of help, like. I really can’t say from the point of view of others what kind of help they need more, like. Well, maybe it really depends on the person and the person’s state of health, that is, how bad she has it like, that, but, but, well, for me, from time to time, I get this attack of … like something like a little neuralgia like, then it’s like, but otherwise I can cope well in everyday life.’* (I3)

Women with IPM need more help at home after childbirth than healthy women. There were problems with holding and transporting the child and doing household chores. The quotes revealed that because of the women’s special needs, health professionals made exceptions to make their lives easier. In addition, various methods, and aids for transporting/holding the child and the service of a personal assistant were used to improve the functioning of everyday life.

### Need for professional help

In this category, the interviewees pointed out the personal need for professional help in pregnancy, childbirth, and the postpartum period. The interviewees shared the opinion that, regarding their disability, they would need more information and support from the midwife both when planning pregnancy and during pregnancy and postpartum period:

*‘In fact, already planning the pregnancy, I would like to get more information from the midwife and doctors (pause), whether there is any hope for someone like me and what kind of difficulties I have to face.’* (I3)

According to one of the interviewees, it was difficult for her during her pregnancy because she had no one to ask for information related to her disability regarding her pregnancy. In addition, she felt embarrassed, being an older first-time mother who is in a wheelchair and needs constant assistance. She would have liked more information to prepare for life with a child and to hear other people’s experiences of how they managed:

*‘And well, maybe also a little that, well, there was no, yes, let’s say something like that, er, (pause, sigh) well, I mean … There was no possibility to ask for more specific advice … well, of course, it’s embarrassing to be at that age, and that’s all, but well, is there something like (sigh) that works due to the fact that you’re in a wheelchair and maybe you need more help yourself so that I can talk about what to prepare for more or read how others are doing, what has been done, what is … people are helpful, what is used and to find out that it is actually positive.’* (I2)

During the interviews, the women were asked whether midwives could have additional knowledge about mobility impairment. The interviewees believed a midwife should have this knowledge to advice women on restrictions due to various physical problems, what to watch for, etc. This would immediately give women information on how to behave and what to monitor or correct if necessary:

*‘I think that it could be yes, in the sense that a woman can also take care of herself, see if she really has back problems or something with her legs; I think there should be.’* (I3)

*‘I was told that they don’t know exactly either and that they have no experience in this field. And that you must research from somewhere.’* (I1)

Based on the quotes from the interviewees, professional help in maternity care must include the provision of broad-based information to support pregnancy planning and pregnancy of a woman with special needs. The participants expected additional knowledge from the midwives regarding the limitations resulting from mobility impairment to receive information on how to alleviate physical problems or ailments during pregnancy.

Childbirth itself is a stressful challenge for a woman with IPM, which requires professional and sensitive help and social support from midwives and doctors. One interviewee had a last-minute discussion with the midwife in the operating room about which form of anesthesia would be used for her:

*‘That everything was completed according to plan and that we also had an operating room for the cesarean section just like we discussed until the very last moment, the midwife said, can it be done, (pause) er, can it be confirmed or can it be done (pause) pause) that, well, anesthesia with a spinal injection … so at the very end, we decided on general anesthesia. But the doctor attending the birth was really very open and understanding. We studied all kinds of possibilities. Is it somehow possible in another way so that we can see if and how it can be done?’* (I2)

The quote highlights that the midwife discussed different pain relief methods for cesarean section while involving the woman, and the doctor’s attitude also indicates that this woman experienced human-centered healthcare. However, women could be told about pain reduction methods as early as possible to avoid excessive tension and to give time for a considered decision:

*‘Even though I had a vaginal delivery, and everything went quickly, I would have liked to know more about birthing positions (pause), maybe there is a more comfortable position for people like us. As ordinary women can choose.’* (I3)

The given quote eloquently shows that a woman with IPM could also choose the birth position if her condition allows it, and the midwife could encourage the choice of position.

During the postpartum period, the interviewees expected more information and instruction from the midwives. When asked what kind of support or help the women would have needed after childbirth, according to one of the interviewees, she would have liked breastfeeding advice from midwives. She expected more understanding and empathic support from the staff. In addition, she needed someone to teach her how to perform childcare activities based on her health status:

*‘Well, the first time, I definitely would have needed breastfeeding counseling. But I didn’t get that. Since I have a friend who is a midwife, I got this help from her (laughter).’* (I1)

*‘I would have liked to know more about aspects of caring for a baby, especially when my (pause) movements are clumsy and so on. What to keep in mind, some useful knowledge or …’* (I2)

The quote show that for the interviewees, postpartum needs were related to the child and its care, but they did not different to a great extent from the needs of women without disabilities. The situation in which the woman did not receive adequate conventional counseling may be due to the fact that midwives are afraid or do not know how to communicate with disabled women. In addition, it may mean that the woman wanted a little more breastfeeding advice based on her disability, but the midwives lack the training to do so.

According to the interviewees, they received enough usual postnatal information, but there was a lack of counseling based specifically on disability:

*‘Yeah, well, the usual. Yes, sure. The usual. It is like most, er, and mothers get their postpartum. That since everything went quite well, nothing really … I think I got a kind of general information quite nicely.’* (I2)

The readiness of midwives in the postpartum department to advice women according to their special needs was considered important. For example, a midwife could teach how to hold a baby correctly and ergonomically in her lap or how to breastfeed more effectively in case of a physical disability. The midwife could have basic information about various aids (pram that can be connected to a wheelchair; adjustable changing table, reclining chair, etc.) for mothers with IMP:

*‘Well, the midwife’s service, well, they could probably have more of that in their preparation, uh, so that they would just learn, for example, breastfeeding techniques to teach … to the mother, different positions, how to hold the baby, rather, as the legs open doesn’t always work, so there are nuances and prepared, that in my case it was, the midwife herself read more on the internet and looked for different ways to teach me, in fact nowadays a midwife should be prepared, that when a woman with special needs comes, that she already knows what advice to give to that person, maybe it is how to breastfeed, maybe the mother has, well, to hold in her arms if it is somehow obstructed. Er … They could also have some information about aids … Well, for example, baby carriages, which are available in some places, can be attached to a wheelchair so that you can walk in the city.’* (I2)

The previous quote gives an idea of what the interviewees expect from a midwife during postpartum. The result suggests that midwives may need additional training in these areas, according to women. It makes the work of midwives easier if they know what women expect from them and how they should develop their own knowledge. In addition, the quote may indicate the need for a health professional to be part of the interdisciplinary team supporting the woman when the need arises.

Women with IPM may have a greater need for help after childbirth in the form of various aids or personal assistance:

*‘Well, well, just the same, just that, uh, just, uh, well, actually, I have a personal assistant next to me, because what will happen, you never know, helps me do various jobs, the assistant was there too, of course, helped when I had the baby, she also helped a lot, a lot, then we were together. We did everything together, actually … Like I didn’t get any extra help.’* (I2)

*‘It would be good if the midwife service was longer for women with special needs like us. I mean just postpartum. All kinds of problems can occur. Well, I think that anyone can help at home, but a midwife can still give professional advice or support.’* (I3)

This quote emphasizes that any kind of help is necessary for a woman with IPM, but the professional help of a midwife is the key factor for more effective coping and ensures better maternity care. Finally, the interviewees expected understanding, emotional support, and relevant information from midwives during pregnancy, childbirth and the postpartum period. They expected the midwives to be able to carry out disability-related counseling.

## DISCUSSION

Although the maternity care experiences of women with IPM were mostly positive, they encountered several obstacles and problems during pregnancy, childbirth, and the postpartum period. There were several areas for improvement like counseling during pregnancy based on mobility disabilities as a special need, specifics related to childbirth, breastfeeding, and childcare, which considers limitations due to disabilities, etc. Women with IPM are different from women without disabilities, and they need additional support and counseling.

### Pregnancy

O’Connor-Terry and Harris^20^ pointed out in their study that women with IPM thought that their disability prevented them from having children and knowing that they were able to conceive was a life-changing process for them, supported by both loved ones and healthcare professionals. The journey to pregnancy for women with IPM who participated in this study varied. Two women had no miscarriages, but one woman had multiple miscarriages and intrauterine fetal death before the pregnancy ended in childbirth. Neither miscarriages nor fetal deaths were associated with disability, which may indicate that the experience of pregnancy is not always related to the presence of a disability. Nevertheless, these experiences may have been traumatic, and to ensure the best maternity care for pregnant women with IPM, the various risks to the health of the mother and the fetus must be considered, regardless of the fact that IPM may not always cause complications. Walsh-Gallagher et al.^4^ found in a study conducted in Ireland that midwives and gynecologists often did not take kindly to the pregnancies of women with disabilities. Contrary to the aforementioned, the women who participated in this study had mainly pleasant experiences with midwives during pregnancy, except for one woman whose midwife did not accept her friend who was brought to the pregnancy appointment as a support person, while the midwife did not have a negative attitude towards pregnancy. The situation was emotionally disturbing for the woman and inhibited the development of a sense of security. The authors of the article agree that women with disabilities may experience different reactions from healthcare workers in healthcare facilities, which can often be related to the prejudices and general attitudes of the workers based on their previous experiences and knowledge rather than the disability. Schildberger et al.^21^ found in a study conducted in Austria that women with disabilities did not receive sufficient support or acceptance from the people around them in relation to pregnancy, childbirth, and motherhood. However, the results of this study revealed that the participating women had a functional support network in the form of both partners and family. Women with IPM often need more support and help than healthy women, so a functioning support network can be decisive in the decision to have children. The study of Mitra et al.^12^ highlights that health professionals face several obstacles in terms of providing maternity care to pregnant women with IPM; for example, they perceive that they lack sufficient training and practice, and because of this, they are uncertain about providing maternity care to women with mobility impairments.

The present study revealed that the interviewees expected additional knowledge from midwives regarding IPM, and one woman perceived that she had no one to ask for information related to mobility impairment from anyone regarding pregnancy. The studies of Mitra et al.^12^ and Smeltzer et al.^13^ show that midwives do not have enough necessary knowledge to share with women with IPM.

In the Estonian context, according to the authors of this study, there is no Estonian-language evidence-based material or guidelines for supporting women with IPM during pregnancy, childbirth, and the postpartum period.

### Childbirth

Tarasoff et al.^3^ point out in their study that women with physical disabilities have a higher probability of cesarean delivery than healthy women. Two out of three women who participated in the present study gave birth by cesarean section and one by vaginal delivery. The interviews did not reveal the exact reason for the cesareans but both were planned, according to the women. Biel et al.^22^ find that due to insufficient practice, healthcare providers feel that cesarean section is a more safely managed delivery method for pregnant women with IPM, even in situations where the woman can deliver vaginally. The women who participated in this study confirmed that they experienced human-centered healthcare during childbirth, where their wishes and abilities were taken into account when choosing the method of delivery. The authors of the study share the view that it is difficult to provide maternity care to women with IPM if there is no deeper knowledge and practice related to a specific condition that causes mobility impairments. However, midwives are empathetic professionals with high learning capabilities who want to offer women a human-centered service and a pleasant pregnancy and childbirth experience, so educating midwives would greatly contribute to improving the quality of the service.

### Postpartum period

Horner-Johnson et al.^23^ found that regardless of the way of delivery, women with disabilities stayed in the hospital one day longer on average than healthy women. In this study, the subjects stayed longer than average in the hospital after giving birth. The reasons were both the prolonged recovery associated with the cesarean section and, in one case, the newborn’s health problems. According to the authors of the study, it is positive that women with IPM can stay longer in the hospital after childbirth, as they need more counseling and support than healthy women. Walsh-Gallagher et al.^4^ found that after giving birth, among women with IPM, the problem was that healthcare workers ignored the special needs of disabled women, did not advise them, and doubted the women’s suitability to become mothers. However, the present study revealed that women with IPM experienced problematic behavior and/or attitudes from social and care workers rather than from midwives.

Byrnes et al.^2^ found that women with disabilities usually need more support and information about breastfeeding after childbirth, and the study of Aavik^24^ showed that maybe it is important to include physiotherapists or occupational therapists when supporting women in the postpartum period, which would ensure better coping for women with IPM. König-Bachmann et al.^9^ observe that successful cooperation between different healthcare professionals helps to ensure the best possible result in providing maternity care to women with IPM. Like previous studies, this study highlighted that women with IPM would have liked breastfeeding advice from healthcare workers, basic information about aids, and more support and understanding after giving birth. This may indicate the need for an interdisciplinary team capable of providing effective and empathetic care based on the needs of a woman with IPM. We agree that maternity care for women with IPM is different from that of women without disabilities and that they need additional support, included emotional support and counseling. An interdisciplinary team, which would include a healthcare worker who specializes in women with IPM, would make working with pregnant women with mobility impairments and with IPM women who have given birth much more efficient^24^. In addition, it would make the maternity care experience more pleasant for women with IPM and would meet their needs.

The women who participated in the study had an increased need for help at home after childbirth. There were mainly problems with transporting the child and performing daily tasks. An important role for women played the techniques learned for moving with a child and various aids. We agree that women with IPM have difficulty finding aids that can be adapted to them. Becker et al.^25^ found that the support of family and healthcare workers, such as a home nurse or a postpartum doula, was important for women with disabilities after childbirth. We also agree that postpartum coping is a more difficult challenge for women with IPM than for healthy women. That is why the support of the midwife, and the support network of loved ones are very important in the lives of these women with IPM, both on a mental and physical level.

### Need for professional help

For the need for professional help, the interviewees summarize important needs both during the journey to pregnancy, during pregnancy and childbirth, and in the postpartum period. Women with IPM are mostly expecting relevant information, and help, emotional support, and disability-related counseling. Mitra et al.^12^, Walsh-Gallagher et al.^4^, Byrnes et al.^2^ and Mitra et al.^26^ emphasize that, in addition to providing specific information and supporting disabled women, midwives need training and education to ensure comprehensive maternity care.

### Strengths and limitations

This is the first study on motherhood and maternity care of women with IPM in Estonia, which deals with women’s experiences with motherhood and becoming a mother. The strength of the study is the fact that primary information is provided from the perspective of women with IPM regarding the experiences of pregnancy, childbirth, and the postpartum period, as well as possible areas for improvement in the provision of maternity care to this vulnerable group, as it is a topic that has not been studied much so far.

The main limitation of the study is the number of participants. It is also necessary to note the limitations arising from the methodology of the study in the formation of the sample (the total number of women with IPM in combination with the time limit of the last pregnancy). Based on the methodology, the fact that the interviews were conducted over the phone, and it was not possible to observe the non-verbal expression characteristics, necessary to the qualitative method, could also have a limiting effect. Despite the limitations, it is important and valuable information that contributes to the provision of more comprehensive maternity care for the vulnerable women with IPM.

## CONCLUSIONS

The research revealed that the experiences of women with IPM are mostly positive. The interviewees were afraid of possible prejudices from the midwives, but the experience was rather the opposite. However, it was felt that midwives lacked disability-based knowledge regarding pregnancy and its aftermath. According to the interviewees, all births were approached in a human-centered way, involving women in decisions related to them. The study revealed that women with IPM stayed in the hospital longer than average after giving birth, and the reasons did not directly stem from mobility impairments. The problems were not experienced by midwives but rather by social worker and care workers, which is why health institutions need to pay attention to this aspect in order to enable a comprehensive human-centered approach to birth mothers and their families during the entire period of hospital stay, regardless of their health status. After childbirth, women’s needs were related to childcare and did not differ significantly from healthy women, but it was still felt that they did not receive enough breastfeeding and childcare counseling. After childbirth, women expected specific advice from midwives based on their special needs, which they did not receive enough of. Therefore, midwives may need additional training in providing more comprehensive maternity care to women with IPM. After giving birth, women with IPM had a greater need for help, both in the form of various aids, techniques, and personal assistance. Similar to pregnancy, a support system was very important for women at home after childbirth. In the future, the topic needs greater attention and improvement in the Estonian context in order to ensure more comprehensive maternity care for women with IPM. It is important to provide midwives with specific training, knowledge, and skills in assisting women with IPM during pregnancy, childbirth, and the postpartum period.

## Data Availability

The data supporting this research are available from the authors on reasonable request.
